# Identification and Validation of Three-Gene Signature in Lung Squamous Cell Carcinoma by Integrated Transcriptome and Methylation Analysis

**DOI:** 10.1155/2022/9688040

**Published:** 2022-09-23

**Authors:** Guanghua Li, Libo Wu, Jiaxing Yu, Siyang Zhai, Hailong Deng, Qiushi Wang

**Affiliations:** ^1^Department of Thoracic Surgery, The Second Affiliated Hospital of Harbin Medical University, Harbin 150000, China; ^2^Department of Thoracic Surgery, The Second Affiliated Hospital of Hainan Medical College, Haikou 570100, China; ^3^Department of Thoracic Surgery, Hailun People's Hospital, Hailun 152300, China

## Abstract

Since DNA methylation (DNAm) is associated with the carcinogenesis of various cancers, this study aimed to explore potential DNAm prognostic signatures of lung squamous cell carcinoma (LUSC). First, transcriptomic and methylation profiles of LUSC were obtained from The Cancer Genome Atlas database (TCGA). DNAm-related genes were screened by integrating DNAm and transcriptome profiles via MethylMix package. Subsequently, a prognostic signature was conducted with the least absolute shrinkage and selector operation (LASSO) Cox analysis. This signature combined with the clinicopathological parameters was then utilized to construct a prognostic nomogram via the rms package. A signature based on three DNAm-related genes claudin 1 (CLDN1), ATP-binding cassette subfamily C member 5 (ABCC5), and cystatin A (CSTA) that were hypomethylated and upregulated in LUSC was constructed. Univariate and multivariate Cox regression analysis suggested that this signature, combined with age and TNM.N stage, was significantly correlated with survival rate. Time-dependent receiver operating characteristics and calibration curves suggested the nomogram constructed with age and TNM.N stage variables could accurately evaluate the 3- and 5-year outcome of LUSC. Finally, the average mRNA and protein expression levels of CLDN1, ABCC5, and CSTA in LUSC were verified to be significantly higher than those in paracancerous tissues. Moreover, silencing CLDN1, ABCC5, and CSTA expressions could significantly reduce the carcinogenesis of the A549 cell line. The DNAm-driven prognostic signature consists of CLDN1, ABCC5, and CSTA incorporated with age and TNM. N stage could facilitate the prediction outcome of LUSC.

## 1. Introduction

Lung cancer is a major cause of mortality associated with tumor diseases in both males and females worldwide, and its 5-year overall survival (OS) is only 14–18% [1]. In the classification of lung cancer, non-small cell lung cancer (NSCLC) is the most common subtype, accounting for >85% of cases, which can be further subdivided into lung adenocarcinoma (LUAD) and lung squamous cell carcinoma (LUSC). LUAD accounts for ∼40% of all cases of NSCLC, while LUSC accounts for ∼30% of all NSCLC cases [2]. With continuous advancements in the research of targeted drugs, progress has been made in the development of clinical treatment strategies for LUAD. However, the field has seen relatively few improvements in the treatment of patients with LUSC [3, 4]. LUSC typically remains asymptomatic during the early stages until it reaches a visible size, or invasion and distant metastases have developed, rendering it too late for further treatment [5]. Screening for reliable prognostic indicators would be beneficial for improved designation of individualized treatment methods for patients with LUSC.

The rapid development of next-generation sequencing and microarray technology has provided great convenience for the analysis of genetic alterations in carcinogenesis and the field of tumor biomarker screening [6]. Various experimental studies have previously revealed specific differentially expressed genes (DEGs) and molecular mechanisms associated with the physiology of LUSC [7–9]. However, neither early diagnostic markers nor prognostic markers for LUSC were considered for further clinical application due to the lack of sensitivity and specificity [10, 11].

DNA methylation (DNAm) is a major epigenetic mechanism that could mediate various biological processes, like cycle progression, proliferation, DNA repair, and tumor development, by silencing the target genes' expressions at the transcriptional level [12]. DNAm results in the formation of stable structures that are easy to detect and has been shown to occur during early tumorigenesis events [13]. Besides, in lung cancer, it was found that DNAm fragments could be detected in peripheral blood and bronchial epithelial exfoliated cells, and had a similar DNAm profile with carcinoma in situ, which meant that the diagnosis and screening of lung cancer could be realized with less invasive diagnostic technology combined with DNAm profile detection [14]. In addition, researchers had realized the significant role of DNAm in lung cancer and have carried out relevant research. For example, Yang et al. explored the methylation differences between LUSC and LUAD, which might be the key clue to explore the pathogenesis of LUAD and LUSC [15]. Jurmeister et al. developed a DNA methylation profiling-based machine learning method to distinguish metastatic head and neck squamous cell carcinoma from primary LUSC [16]. These previous studies suggested that the potential value of applying methylation profiles in LUSC was gradually recognized.

Therefore, a prognostic model based on DNAm-related genes may be beneficial for the clinical intervention of LUSC. In the study, the DNAm and transcription expression profiles obtained from The Cancer Genome Atlas (TCGA) database were integrated to screen for the DNAm-driven signatures in LUSC. This signature was then used in conjunction with the clinicopathological risk factors to establish a nomogram to predict the OS of patients with LUSC. And the DNAm-driven signatures would be expected for the application of prognostic evaluation and personalized treatment of LUSC patients.

## 2. Materials and Methods

### 2.1. Data Collection

The mRNA-sequencing profiles of the 501 LUSC samples and 49 paracancerous tissues samples, in addition to corresponding follow-up demographic features, were both harvested from the TCGA database (TCGA-LUSC, https://portal.gdc.cancer.gov/projects/TCGA-LUSC). In addition, a separate cohort of LUSC cohort, which contained 130 samples with complete prognostic information, was also downloaded from UCSC Cancer Genomics Browser (https://xenabrowser.net/) for the validation cohort.

### 2.2. DEG Screening between LUSC and Paracancerous Tissues

Potential DEGs between the 501 LUSC samples and 49 paracancerous lung tissues were screened with the “DESeq2” package [17] from *R* software (version 3.5.1) with the cut-off criteria set to |log_2_ fold change (FC)| >1 and adjusted *P* value <0.05.

### 2.3. Screening for DNAm-Related Genes in the LUSC Samples

DNA methylation profiles (370 LUSC and 42 paracancerous lung tissues) were harvested from the TCGA database [18]. The methylation level was represented by *β*-values ranging from 0 to 1 (the ratio of the methylation probe vs total probe intensities) [19]. Next, the DNAm-related genes were screened with MethylMix 2.0 in *R* by analyzing whose methylation alterations were associated with gene expression [20]. Lastly, the DNAm-related genes were screened according to the principle of negative correlation between the levels of DNA methylation and the expression of its corresponding mRNA using the cut-off correlation (cor) value of <−0.3 and adjusted *P* values <0.05. Differential DNAm levels were identified using the Wilcoxon rank-sum test with the threshold of adjusted *P* values set at <0.05 between 370 LUSC and 42 paracancerous lung tissues [21].

### 2.4. Functional Gene Enrichment Analysis

For the exploration of potential biological effects mediated by the screened DNAm-related genes, Gene Ontology (GO) enrichment analysis was conducted using the “clusterProfiler” package (v3.11.0) [22]. In addition, Gene Set Enrichment Analysis (GSEA) analysis was conducted using the GSEA-3.0.jar program (http://www.gsea-msigdb.org/gsea/index.jsp) to investigate the potential signaling pathways involved by referencing the Molecular Signatures Database of c2 [23]. Results with *P* values <0.05 were considered to be significant.

### 2.5. Development of the DNAm-Driven Signature for Survival Prediction

Kaplan-Meier (K-M) analysis by the log-rank test was first conducted to screen the DNAm-related genes that could be correlated with the prognosis of LUSC. Subsequently, Least Absolute Shrinkage and Selection Operator (LASSO) Cox regression were utilized to narrow the list of candidate DNAm-related genes [24]. A prognostic signature score was calculated using the linear combination of the regression coefficient harvested from the mRNA expression levels of the interested genes multiplied by the multiple Cox regression model. Each sample can obtain a risk score based on the prognostic signature. Risk score was transferred to *z*-score and samples were divided into low- (*z*-score <0) or high-risk (*z*-score >0) groups according to the cut-off*z*-score = 0.

### 2.6. Nomogram Construction and Evaluation

The univariate prognostic factors for OS were determined using Cox regression analysis with the TCGA LUSC cohort followed by subsequent multivariate analysis with the threshold of two-sided*P* value <0.05. Next, the prognostic signature was integrated to map and construct a prognostic nomogram to accurately predict the 3- and 5-year OS of LUSC. To evaluate the accuracy of the nomogram, internal validation (1,000 bootstrap resamples) was performed to assess the fit degree, which was indicated by the calibration diagrams [25]. Time-dependent receiver operating characteristics (ROC) curves were also constructed to evaluate the prognostic role of this nomogram for predicting the 3 and 5-year OS.

### 2.7. Collection of Tissue Samples

All human tissues involved in the present study were collected and preserved in The Second Hospital of Harbin Medical University (Harbin, China). LUSC samples (*n* = 15) were collected in the operation from patients with pathologically confirmed LUSC who had no other systemic diseases and did not receive radiotherapy, chemotherapy, allogeneic blood transfusion, or cellular immunotherapy before operation. The demographic characteristics of these patients who were voluntary contributions to LUSC samples, including age, sex, clinical tumor stage (TNM), and the presence of distant metastasis are presented in Table 1. The collected tissues were rinsed with sterile normal saline and were divided into 3 parts for RT-qPCR, western-blot, and histopathology examination. For RT-qPCR and western-blot assay, the tissues were quickly placed in a cryotube, frozen in liquid nitrogen, and stored in a −80°C low-temperature freezer. As for histopathology examination, the tissues were immersed in 4% paraformaldehyde overnight, followed by dehydrated in xylene and gradient alcohol solutions, embedded in paraffin. The present study was approved by the Ethics Committee of The Second Affiliated Hospital of Harbin Medical University (approval no. 20-006) and all patients provided written informed consent.

### 2.8. RNA Extraction and Reverse Transcription-Quantitative PCR (RT-qPCR)

The steps of RT-qPCR assay were referred to reported study [26]. The extraction of total RNA from all the samples was performed with TRIzol® reagent (Invitrogen, USA) according to the instrument. The extracted RNA quality was evaluated by the A260/A280 ratio with NanoDrop® 2000 spectrophotometer (Thermo Fisher Scientific, USA). And the A260/A280 ratio of all samples was between 1.9 and 2.1. All sample concentrations were between 1300 and 2000 ng/*μ*l. cDNA was synthesized from a total of 1 *μ*g RNA with PrimeScript™ RT Reagent (Takara Bio, Japan) in line with the manufacturer's instrument. RT-qPCR was conducted using the FastStart Universal SYBR Green PCR Master Mix kit (Roche, Cat.No 4913914001) with thermocycling conditions of initial heat-activation at 95°C for 10 min, followed by 45 cycles of 95°C for 15 sec, 60°C for 30 sec and 72°C for 30 sec. The primers sequences for qPCR are provided in Table 2 and GAPDH was utilized as the internal control. Gene expression levels were normalized relative to that of GAPDH using the 2^−ΔΔ*Cq*^ method [27].

### 2.9. Protein Extraction and Western-Blot Assay

The total protein from all samples was harvested with the RIPA reagent (Beyotime Institute of Biotechnology), which contained 1 mM PMSF as a reported study [26]. After the protein concentration was measured using a BCA Kit (Beyotime Institute of Biotechnology), 40–80 *μ*g protein per lane was separated using 15% SDS-PAGE, followed by transfer to PVDF membranes (MilliporeSigma). Next, the membranes were incubated with primary antibodies against claudin 1 (CLDN1; Cat.No. #13995. Dilution 1 : 1,000; Cell Signaling Technologies, Inc.), ATP-binding cassette subfamily C member 5 (ABCC5; Cat.No. ab230674. Dilution 1 : 2000; Abcam) and cystatin A (CSTA; (Cat.No. ab166805. Dilution 1 : 1,000; Abcam) overnight at 4°C. And the membranes were washed with 0.05% Tween-20 (TBST) three times, followed by incubation with HRP-conjugated secondary antibodies for 1 h at room temperature. Finally, an enhanced chemiluminescence detection system (Bio-Rad Laboratories, Inc.) was utilized to detect the protein bands.

### 2.10. Immunohistochemistry Staining

To verify the presence of CLDN1, ABCC5, and CSTA expression in the LUSC samples, the collected samples were embedded in paraffin, followed by sectioning into 3-*μ*m thick sections following the reported study [26]. Primary antibodies for CLDN1 (Cat.No. #13995. Dilution 1 : 200; Cell Signaling Technologies, Inc.), ABCC5 (Cat.No. ab230674. Dilution 1 : 100; Abcam) and CSTA (Cat.No. ab166805. Dilution 1 : 200; Abcam) were utilized to incubate the slides overnight. HRP-conjugated secondary antibodies were then used to incubate the slides for 1 h at room temperature. Next, the slides were visualized using 3,3′-diaminobenzidine for 5 min and the nucleus was counterstained with hematoxylin. Finally, images were collected using the Olympus BX51 light microscope (Olympus Corporation).

### 2.11. Cell Culture and Treatment

NSCLC cell line A549 was harvested from the Chinese Academy of Sciences (Shanghai, China) and cultured in RPMI-1640 medium (Hyclone, USA) with 10% fetal bovine serum (FBS, TIANHANG, China) in 5% CO_2_, 37°C incubators. The short interfering RNA (siRNA) for CLDN1 (sequence: 5′-3′, GCTGAATCTGAGCAGCACATT), ABCC5 (sequence: 5′-3′, GCAGTACAGCTTGTTGTTAGT) and CSTA (sequence: 5′-3′, AGGTACGAGCAGGTGATAATA) were obtained from GenePharma (Shanghai China). The cell transfection was performed with Lipofectamine® 3000 (Invitrogen, USA) according to the instructions. And the transfected cells were continued to culture for 48 h, followed by the subsequent experiments. The cell without any treatment named Control and cells were accepted siRNA transfection named siCLDN1, siABCC5, and siCSTA, separately.

### 2.12. Cell Proliferation Experiment

In order to evaluate the effects of CLDN1, ABCC55, and CSTA genes on the proliferative ability of A549 cells, the groups of Control, siCLDN1, siABCC5, and siCSTA cells underwent in the 5-ethynyl-2′-deoxyuridine (EdU) assay via the EdU kit (Solarbio, Chian) following the manufacturer's protocol. Images were obtained under a fluorescence microscope (Leica, Germany) and analyzed with the ImageJ software.

### 2.13. Colony Formation Assay

In order to evaluate the effects of CLDN1, ABCC5, and CSTA genes on the clonogenic ability of A549 cells, the colony formation assay was conducted on the groups of Control, siCLDN1, siABCC5, and siCSTA cells following the method of a previous study [28]. And the colonies (>50 cells) were counted and analyzed via ImageJ.

### 2.14. Transwell Assay

In order to evaluate the effects of CLDN1, ABCC55 and CSTA genes on the invasive ability of A549 cells, the transwell assay was conducted on the groups of Control, siCLDN1, siABCC5, and siCSTA cells following the method of a previous study [28]. The images were harvested via an optical microscope (Olympus, Japan) and analyzed via ImageJ.

### 2.15. Statistical Analysis

The bioinformatics analysis in this study was supported by Sangerbox platform [29]. All statistical analysis was performed using in the *R* software (version 3.5.1). And the results were presented as the mean ± SD. The survival analysis was performed via the Kaplan–Meier method and analyzed by the log-rank test. The paired Student's *t*-test was utilized for comparison between the two groups. Each sample was detected three times in RT-qPCR and Western-blot assays. *P* < 0.05or an adjusted *P* value <0.05 was considered to indicate a statistically significant difference.

## 3. Results

### 3.1. DEGs Identification between LUSC and Nontumorous Lung Tissues

First, the DEGs between LUSC and paracancerous tissues under the threshold of adjusted *P* value <0.05 and |log_2_FC| >1 were identified. There was a total of 806 DEGs, including 327 upregulated and 479 downregulated genes (Figure S1).

### 3.2. DNAm-Related Genes Identification

**Figure 1:**
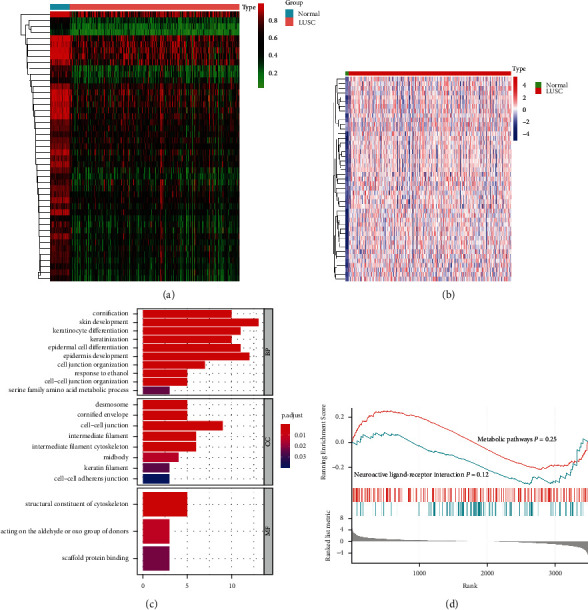
Screening for the DNAm-related genes. Heatmap for the (a) DNAm and (b) mRNA levels of the screened DNAm-related genes between LUSC and paracancerous samples. (c) GO enrichment analysis and (d) GSEA analysis of the 45 DNAm-related genes. DNAm, DNA methylation; LUSC, lung squamous cell carcinoma; GO, Gene Ontology; GSEA, gene set enrichment analysis.

Subsequently, genes with a negative correlation between their mRNA and corresponding DNAm levels were considered to be DNAm-related genes. Under the thresholds of adjusted *P* value <0.05 and cor value <−0.3, a total of 45 hypomethylated DNAm-related genes were identified (Table SI). The DNAm levels of the 45 screened DNAm-related genes are shown in Figure 1(a) while their corresponding mRNA expression levels are shown in Figure 1(b).

### 3.3. Functional Exploration of DNAm-Related Genes

Next, with the goal of investigating the potential biological effect of the screened DNAm-related genes, GO and GSEA enrichment analyses were performed. In the Biological Process (BP) category, these genes were mainly involved in the “cornification,” “skin development,” “keratinocyte differentiation,” “epidermal cell differentiation” and “epidermis development.” In the Cellular Component (CC) category, the genes were enriched in “desmosome,” “cornified envelope,” “cell-cell junction,” “intermediate filament” and “intermediate filament cytoskeleton.” For the Molecular Function (MF) category, there were only three terms of “structural constituent of cytoskeleton,” “acting on the aldehyde or oxo group of donors” and “scaffold protein binding” that were enriched (Figure 1(c)). There was no significance in any of the signaling pathways enriched, but terms of “Metabolic pathways” and “Neuroactive ligand-receptor interaction” were the only two mechanism pathways where the DNAm-related genes were involved (Figure 1(d)).

### 3.4. Screening and Establishment for DNAm-Related Signature Associated with Prognosis

**Figure 2:**
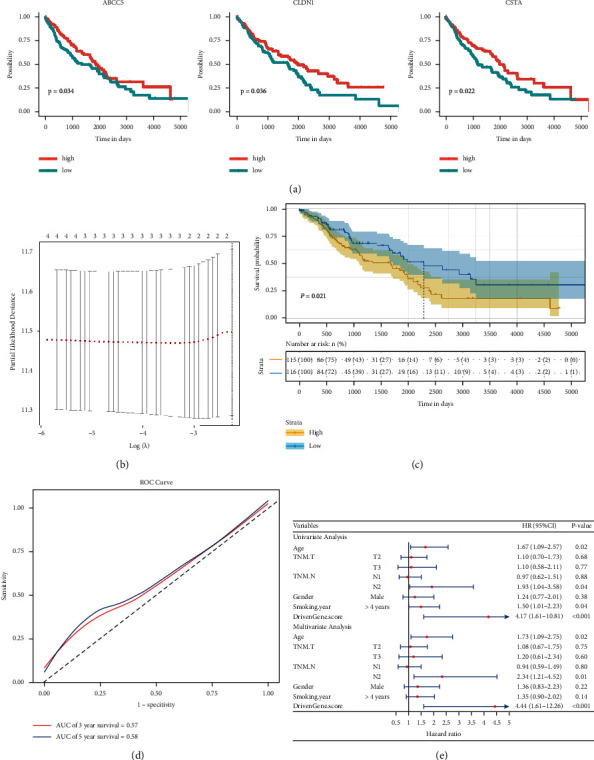
Screening and establishment for DNAm-driven signature associated with prognosis. (a) Survival analysis for CLDN1, ABCC5, and CSTA expressions in LUSC. (b) A 10-foldcross-validation for tuning parameter selection in the Least Absolute Shrinkage and Selection Operator model. Partial likelihood deviance is plotted against log (*λ*), where *λ* is the tuning parameter. Dotted vertical lines were drawn at the optimal values by minimum criteria and 1-s.e. criteria. (c) Comparison of OS between the high-risk and low-risk score groups. (d) Time-dependent receiver operating characteristic analysis was performed to evaluate the accuracy of the prognostic signature. (e) Univariate and multivariate regression analysis was performed to identify significantly predictive factors associated with OS. OS, overall survival (days); CLDN1, Claudin 1; ABCC5, ATP-binding cassette subfamily C member 5; CSTA, Cystatin A.

First, the DNAm-related genes related to prognosis were screened by K-M analysis. With the *P* < 0.05 criterion from the log-rank test, ABCC5, CLDN1, and CSTA were associated with a superior prognosis in LUSC (Figure 2(a)). Next, the three-gene-based classifier screened using the LASSO Cox regression model was constructed using the specification of the tuning parameter meeting the criteria of minimal partial likelihood deviance (Figure 2(b)). The coefficients of the ABCC5, CLDN1, and CSTA genes were calculated to be −0.0075, −0.0581, and −0.0477, respectively. Therefore, the LASSO Cox-derived signature score was calculated as follows: DrivenGene score = (−0.0075^*∗*^ ABCC5 mRNA level) + (−0.0581^*∗*^ CLDN1 mRNA level) + (−0.0477^*∗*^ CSTA mRNA level). And the LUSC patients in TCGA samples were divided into high-risk and low-risk subgroups based on whether their DrivenGene scores were higher than the score median or not. K-M analysis suggested that the DNAm-driven signature could significantly distinguish patients with different prognoses (Figure 2(c)). Time-dependent ROC curves demonstrated a moderate efficacy of the signature on predicting the 3- and 5-year OS, with area under the curve (AUC) values of 0.57 and 0.58, respectively (Figure 2(d)). Log-rank univariate analysis revealed that the signature and the patient age at diagnosis, N2 stage in the TNM staging system, and smoking year were significantly associated with OS (Figure 2(e)). These variables, excluding the variable of smoking year, were also found to be significant according to multivariate Cox regression analysis (Figure 2(e)). These results suggest a role of this independent signature in predicting OS in patients with LUSC (hazards ratio, 4.44; 95% confidence interval, 1.61–12.26).

### 3.5. Nomogram Construction and Evaluation of OS for LUSC

**Figure 3:**
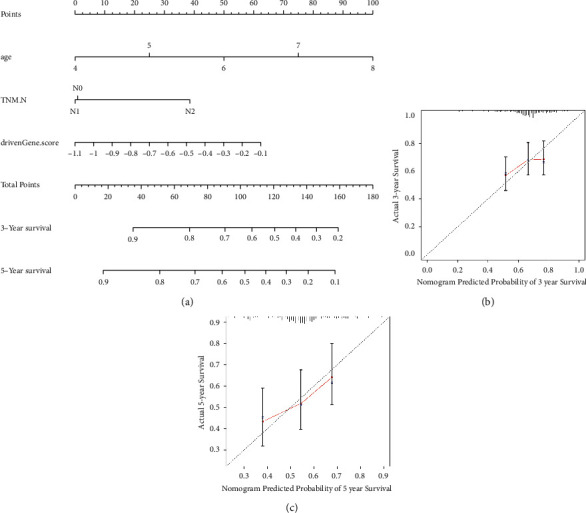
Nomogram generation and validation. (a) The constructed nomogram for evaluating the 3- and 5-year OS of patients with lung squamous cell carcinoma. Calibration curve of (b) 3- and (c) 5-year OS for the nomogram. The black dotted line represents the ideal predictive model, whilst the red solid line represents the observed model. OS, overall survival (days).

The factors (age, TNM.N staging, and signature) that were found to be significantly correlated with the outcome were included in the nomogram construction procedure for predicting or evaluating the 3-year and 5-year outcome of LUSC (Figure 3(a)). In the nomogram, the signature contributed moderately to the prediction of OS. In addition, calibration plot diagrams demonstrated a reasonable consistency with the predicted model and the experimentally observed model of the 3- and 5-year OS (Figures 3(b) and 3(c)).

### 3.6. External Validation for Signature Prognostic Nomogram

**Figure 4:**
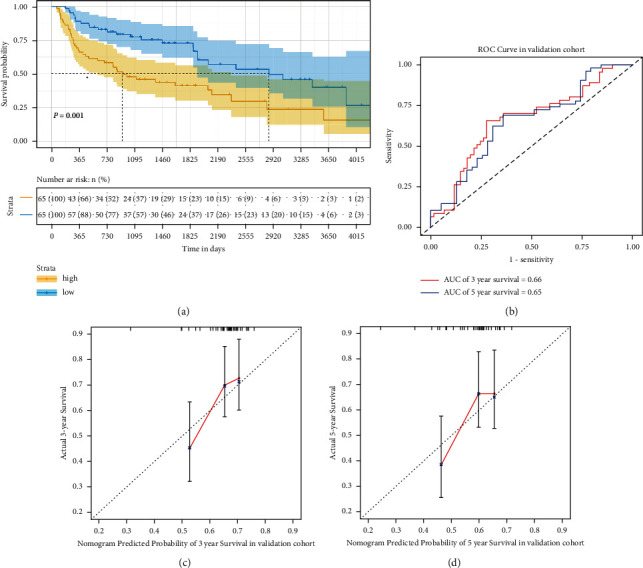
External validation of the prognostic signature and nomogram for LUSC. (a) Comparison of OS between the high-risk and low-risk score groups in the validation cohort. (b) Time-dependent receiver operating characteristic analysis for evaluating the accuracy of the prognostic signature in the validation cohort. Calibration curve of the (c) 3- and (d) 5-year OS for the nomogram in the validation dataset. LUSC, lung squamous cell carcinoma; OS, overall survival (days).

A validation cohort of LUSC from the UCSC Cancer Genomics Browser was selected to validate the reliability of the constructed prognostic signature and nomogram. In the results, the signature could significantly distinguish patients in the validation cohort with different prognoses as well. The OS of the high-risk group was significantly shorter in comparison with that of the low-risk group according to the K-M analysis (Figure 4(a)). The AUC values from the time-dependent ROC curves were 0.66 and 0.65 for 3- and 5-year OS, respectively (Figure 4(b)). The calibration curves of the nomogram for the possibility of 3- and 5-year OS showed accurate predictive ability in the validation cohort (Figures 4(c) and 4(d). These results suggest that this prognostic signature and nomogram performed well both in the training and validation cohort used in the present study.

### 3.7. Verification of ABCC5, CLDH1, and CSTA Expression in LUSC and Paracancerous Tissues

**Figure 5:**
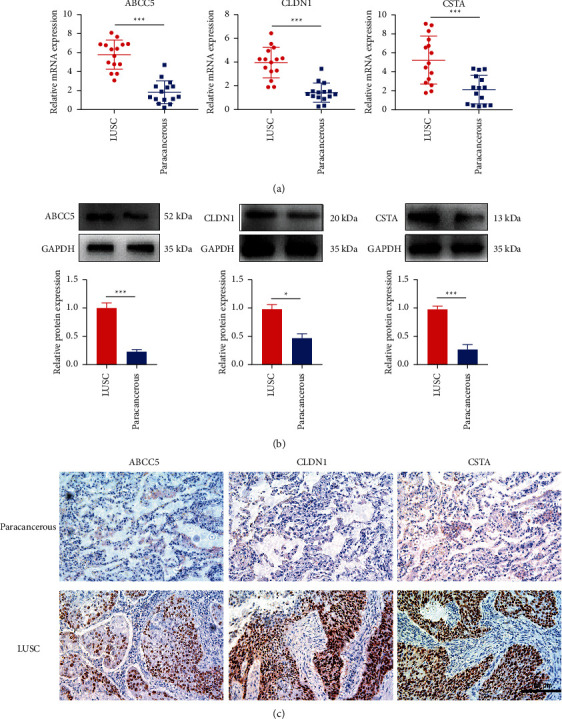
ABCC5, CLDN1, and CSTA expression in LUSC and paracancerous tissues. (a) mRNA expression levels of ABCC5, CLDN1, and CSTA in LUSC and paracancerous tissues. (b) Protein expression levels of ABCC5, CLDN1, and CSTA in LUSC and paracancerous tissues. (c) Representative images of immunohistochemistry staining for ABCC5, CLDN1, and CSTA in LUSC and paracancerous tissues. Magnification, ×200. Data are displayed as the mean ± standard deviation. Student's *t*-test was performed.^*∗*^*P* < 0.05 and ^*∗∗∗*^*P* < 0.001. LUSC, lung squamous cell carcinoma; CLDN1, Claudin 1; ABCC5, ATP-binding cassette subfamily C member 5; CSTA, Cystatin A.

In total, 15 pairs of cancer and adjacent noncancerous tissues were collected for molecular and histochemical validation. In the results, the mRNA expression levels of ABCC5, CLDH1, and CSTA were higher in LUSC compared with those in the paracancerous tissues (Figure 5(a)). The trend in the protein expression levels of ABCC5, CLDH1, and CSTA were consistent with those of their corresponding mRNA expression levels, in that they were significantly higher in LUSC compared with those in the paracancerous tissues (Figures 5(b) and 5(c)).

### 3.8. Inhibition of ABCC5, CLDH1, and CSTA Reduced the Carcinogenesis of NSCLC Cells

**Figure 6:**
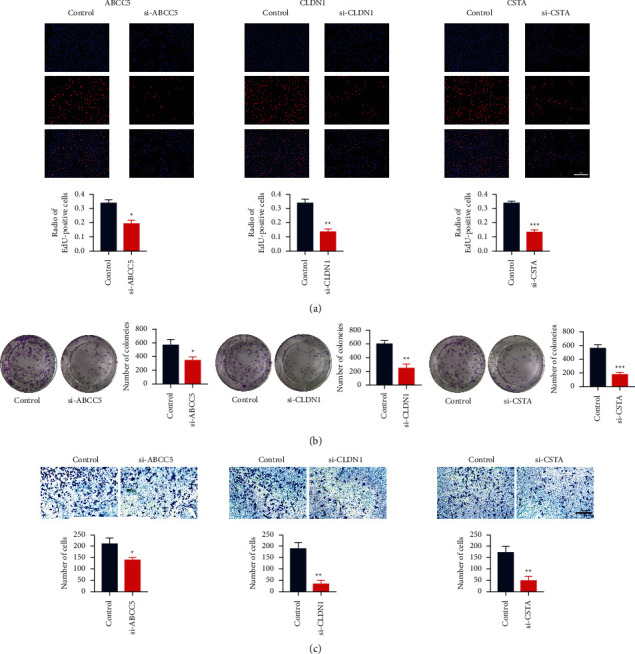
Inhibition of ABCC5, CLDH1, and CSTA reduced the carcinogenesis of NSCLC cells. (a) Representative images of EDU staining for Control, siABCC5, siCLDN1, and siCSTA groups. Magnification, ×200. (b) Comparison of colonies number among control, siABCC5, siCLDN1, and siCSTA groups. (c) Representative images of invasive ability comparison among Control, siABCC5, siCLDN1, and siCSTA groups. Magnification, ×100. Data are displayed as the mean ± standard deviation. Student's *t*-test was performed. ^*∗*^*P* < 0.05, ^*∗∗*^*P* < 0.01, and ^*∗∗∗*^*P* < 0.001. NSCLC, non-small-cell lung cancer; EDU, 5-ethynyl-2′-deoxyuridine; ABCC5, ATP-binding cassette subfamily C member 5; CLDN1, Claudin 1; CSTA, Cystatin A.

In order to investigate whether ABCC5, CLDH1, and CSTA were involved in the carcinogenicity of LUSC, we selected the NSCLC cell line A549 to conduct a series of phenotypic tests. In Figure 6(a), silencing ABCC5, CLDN1 and CSTA could significantly inhibit the proliferative ability of cells. Besides, silencing ABCC5, CLDN1 and CSTA could significantly reduce the number of cellular colonies (Figure 6(b)), as well as the invasive cell number (Figure 6(c)). These results suggested that the screened DNAm-related genes had an effect on the carcinogenesis of NSCLC cells.

## 4. Discussion

At present, LUSC is a leading cause of cancer mortality worldwide [1]. Due to the lack of sensitive and specific biomarkers, a substantial proportion of patients with LUSC cannot receive appropriate treatment, resulting in poor outcomes [3]. TNM staging is currently the most important tool for assessing the prognosis of patients with cancer, including LUSC [30]. However, with the development of molecular diagnostic techniques, their simple and fast nature gradually highlights their value in the diagnosis of early disease, which has been proposed to serve as an effective supplement to TNM staging to improve the accuracy of prognosis evaluation further [25]. Indeed, a large number of markers for LUSC have been previously found, including centrosomal protein 55 [31], integrin subunit *α*11 [32], long interspersed nuclear element-1 [33], and gasdermin D [34]. However, due to the heterogeneity that exists in LUSC tumors, any single marker alone is not sufficient to accurately diagnose or predict the prognosis of this disease [35]. Therefore, the integration of several prognostic markers and clinicopathological parameters nomograms would be able to evaluate the prognosis of patients with cancers more reliably and accurately [36].

In recent years, epigenetic abnormalities have been reported to exert key roles in the carcinogenesis in various cancer, including LUSC [19, 21]. Notably, DNA methylation, which is mediated by DNA methyltransferases by adding a methyl (CH3) group to the fifth position of cytosine, is one of the most extensively studied epigenetic mechanisms in cancer research [37]. DNA methylation tends to be more stable, easier to detect, and reversible during the early stages of tumors [38]. Therefore, it has potential as predicated or therapeutic target for some diseases. In our study, 45 genes were identified that were significantly hypomethylated in LUSC according to the integrative analysis of the transcriptomic and methylation profiles based on data obtained from TCGA. Among the 45 DNAm-related genes, the top five genes with the most significant correlation between methylation level and mRNA expression level were GCLC (Cor = −0.74), ARTN (Cor = −0.73), ABCC5 (Cor = −0.67), FSCN1 (Cor = −0.67) and CA12 (Cor = −0.67), respectively. GCLC is a catalytic subunit of glutamate-cysteine ligase which is responsible for glutathione synthesis [39]. Although ARTN is a member of glial cell-derived neurotrophic factors, the main function of ARTN is to drive migration [40]. ABCC5 is one of the ATP-binding cassette transporters and its main function is to transport a variety of compounds on the cell membrane. FSCN1 is responsible for the formation of actin-based cellular protrusions and is involved in cell migration, adhesion, and cellular interactions [41]. And CA12 as a member of zinc metalloenzymes is mainly participated in extracellular microenvironment pH adjustment and ion exchange [42]. These gene functions suggested that the pathogenesis of LUSC involved various aspects of biological metabolism.

Next, in order to explore the potential biological functions of DNAm-related genes, the GO enrichment analysis was performed. The results revealed that the DNAm-related genes were found to be mainly involved in the “cornification,” “desmosome” and “structural constituent of cytoskeleton” processes, but were not revealed to be significantly associated with any signaling pathways. Cornification is a form of apoptosis that frequently occurs in the squamous cells of epithelial tissues [43]. Zhou et al. previously revealed that cornification would be beneficial in eliminating squamous cancerous cells in LUSC [43]. Desmosomes are forms of cell-cell junction structures that could inhibit the migration, proliferation, and invasion of different tumors. Dysregulation of desmosomal proteins was found in various cancers [44]. In NSCLC, desmosomal proteins plakophilin 1 and DSC1 were previously verified to be associated with tumor development and prognosis [44, 45]. Similarly, the cytoskeleton regulates cell migration, cell division, intracellular transport, and signaling processes. In general, aberrant expression and mutations in cytoskeletal proteins have been found to be associated with tumor cell metastasis [46]. A previous study has shown that glucose-regulated protein 78 secreted by tumors can promote the migration and chemoattraction of macrophages by promoting cytoskeleton remodeling [47]. Therefore, results from the present study suggest that these 45 DNAm-related genes can regulate the development of LUSC through the mechanism of cornification, desmosomal regulation, and structural construction of the cytoskeleton.

Among the 45 DNAm-related genes, CLDN1, ABCC5, and CSTA expressions were positively associated with superior prognosis in patients with LUSC. Subsequently, a novel three-gene (CLDN1, ABCC5, and CSTA) signature to forecast LUSC outcome was established using Lasso-Cox regression. The prognosis signature was found to be an independent prognostic factor for LUSC, where patients in the high-risk group had significantly more poor prognoses compared with that in the low-risk groups. Subsequently, a nomogram was constructed using this three-gene signature and clinicopathological parameters (age and TNM N-staging). AUC values and calibration curves were used to validate this three-gene signature, which confirmed its accuracy for evaluating the 3- and 5-year OS of LUSC. In addition, its reliability was also confirmed in an external UCSC dataset. Finally, in the clinically collected LUSC samples, the expression of CLDN1, ABCC5, and CSTA was found to be upregulated in LUSC compared with that in the paracancerous tissues.

CLDN1 belongs to the claudin protein family, the dysfunction or aberrant expression of which was previously found in various tumors. In esophageal squamous carcinoma, CLDN1 was revealed to promote tumor development and invasion via autophagy activation through the 5′AMP-activated protein kinase/STAT1/Unc-51 like autophagy activating kinase (ULK1) signaling pathway [48]. In addition, CLDN1 was also found to be associated with the malignancy of hepatocellular carcinoma [49], cervical cancer [50], breast cancer [51], and gastric cancer [52]. In lung cancer, CLDN1 expression was reported to promote the chemotherapeutic resistance of NSCLC through ULK1 phosphorylation, but methylation of the CLDN1 gene can enhance the efficacy of chemotherapy to inhibit the progress of LUAD [53]. In the present study, the CLDN1 methylation level was found to be lower in LUSC compared with that in normal tissues, which was consistent with previous studies. ABCC5 is an important member of the ATP-binding cassette transporters (ABC) family, where its main function is to transport a variety of compounds on the cell membrane. Previous reports verified overexpression of ABCC5 can increase the malignancy of prostate cancer [54]. In addition, biological effects of ABCC5 in breast cancer [55], colon cancer [56], pancreatic cancer [57], and nasopharyngeal carcinoma have also been reported [58]. It was found that higher expression of ABCC5 can decrease the sensitivity of the NSCLC cell line to gemcitabine [59]. CSTA belongs to the cystatin protein superfamily, which is mainly expressed in epithelial and lymphoid tissues. Its main function is to protect cells from the hydrolysis of cytoskeletal and cytoplasmic proteins by cathepsins B, H, and L. CSTA has been documented to serve a suppressive role in esophageal squamous cell carcinoma and in lung cancer cell lines [60]. In the present study, the expression of CSTA was higher in LUSC compared with that in paracancerous tissue, where it functioned as a carcinogenic gene for LUSC. Moreover, silencing CLDN1, ABCC5, and CSTA expression could significantly reduce the proliferative, cloning, and invasive abilities of NSCLC cell line. These findings suggested that these three genes were reliable markers or targets for the prognostic prediction or treatment of LUSC.

In conclusion, the results from the present study supported the hypothesis that genes regulated by DNAm could be associated with the prognosis of LUSC. CLDN1, ABCC5, and CSTA-based signature, combined with the clinicopathological parameters of age and TMN N stage, could be utilized as an accurate predictor of LUSC prognosis in clinical practice in the future.

## Figures and Tables

**Table 1:** Clinicopathologic characteristics of LUSC patients.

Age	
Mean ± S.D.	58 ± 8

Sex (%)	
Male	80 (12/15)
Female	20 (3/15)

Tumor size (%)	
T1–2	66.7 (10/15)
T3–4	33.3 (5/15)

Nodal metastasis (%)	
Present	53.3 (8/15)
Absent	46.7 (7/15)

Distant metastasis (%)	
Present	20 (3/15)
Absent	80 (12/15)

TNM stage (%)	
I–II	66.7 (10/15)
III–IV	33.3 (5/15)

**Table 2:** Primers sequences used in the qRT-PCR experiments.

Gene	Forward primer (5′- 3′)	Reverse primer (5′- 3′)
CLDN1	CCTCCTGGGAGTGATAGCAAT	GGCAACTAAAATAGCCAGACCT
ABCC5	AGTCCTGGGTATAGAAGTGTGAG	ATTCCAACGGTCGAGTTCTCC
CSTA	AAACCCGCCACTCCAGAAATC	CACCTGCTCGTACCTTAATGTAG
GAPDH	CCACTCCTCCACCTTTGAC	ACCCTGTTGCTGTAGCCA

## Data Availability

The datasets used and/or analyzed during the current study are available from the corresponding author upon reasonable request.

## Supplementary Materials

Supplementary Materials
*Figure S1*. The heatmap for the DEGs between LUSC and paracancerous tissues. LUSC, Lung squamous cell carcinoma. *Table SI*. List of DNAm-driven genes in LUSC.
