# Supplementary material for: Identification and Validation of Three-Gene Signature in Lung Squamous Cell Carcinoma by Integrated Transcriptome and Methylation Analysis
Source: J Oncol. 2022 Sep 23;2022:9688040. doi: 10.1155/2022/9688040 (PMC9525794; doi:10.1155/2022/9688040)
Supplement: Supplementary Materials — Figure S1. The heatmap for the DEGs between LUSC and paracancerous tissues. LUSC, Lung squamous cell carcinoma. Table SI. List of DNAm-driven genes in LUSC. [file 9688040.f1.zip › Supplementary materials.docx]

**Figure S1. The heatmap for the DEGs between LUSC and paracancerous tissues.** LUSC, Lung squamous cell carcinoma.


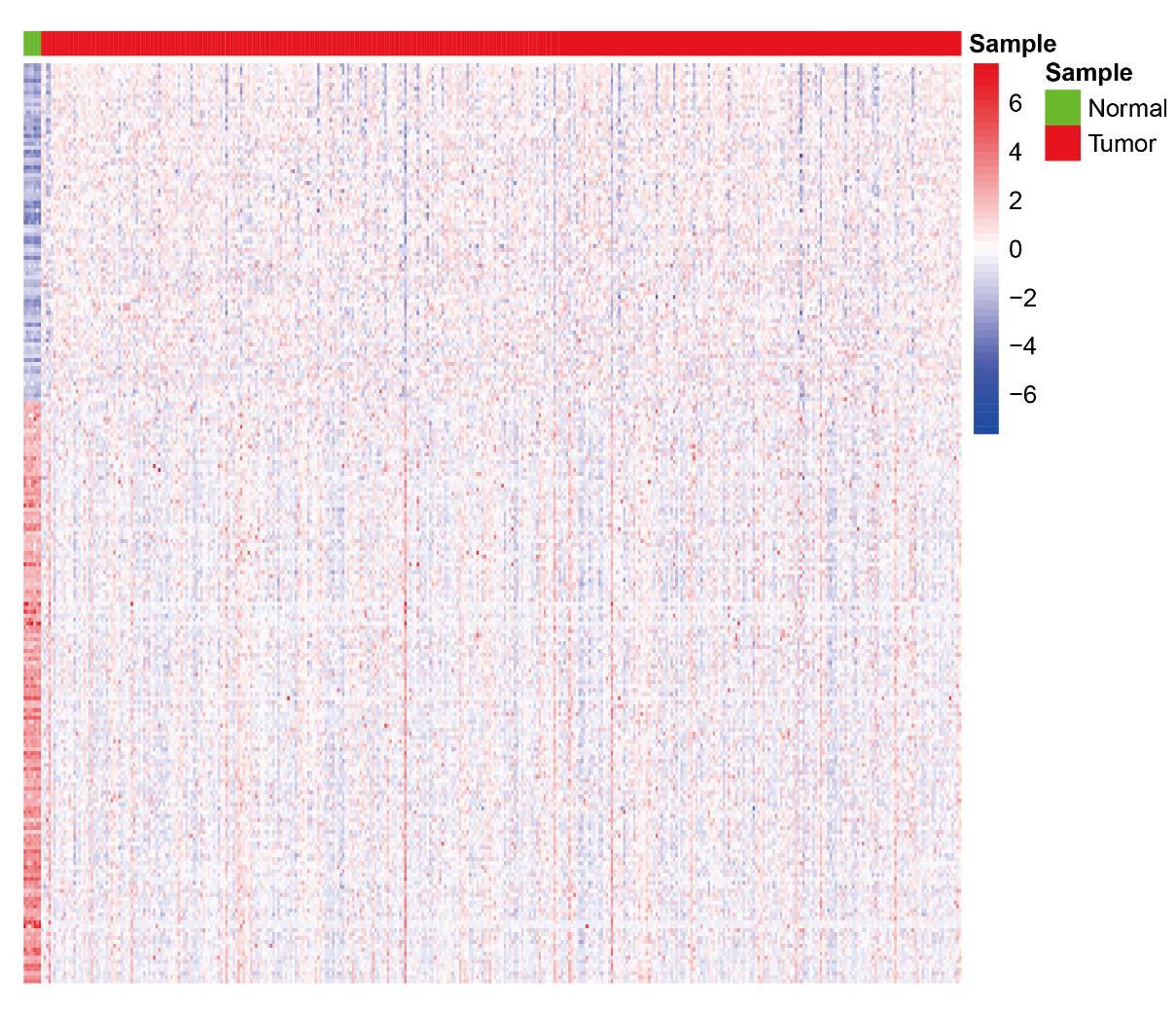


Table SⅠ List of DNAm-Driven Genes in LUSC

| symble | function | normalMean | tumorMean | logFC | cor | corPavlue | adjustP.value |
| --- | --- | --- | --- | --- | --- | --- | --- |
| GCLC | Glutamate-cysteine ligase catalytic subunit | 0.94 | 0.69 | -0.25 | -0.74 | 8.95E-66 | 5.12E-19 |
| ARTN | A member of the glial cell-derived neurotrophic factor | 0.81 | 0.57 | -0.24 | -0.73 | 1.62E-62 | 4.09E-22 |
| ABCC5 | A member of ATP-binding cassette transporters. | 0.7 | 0.32 | -0.38 | -0.67 | 2.41E-49 | 1.12E-20 |
| FSCN1 | Regulatory factor of actin-based cellular protrusions formation. | 0.65 | 0.42 | -0.23 | -0.67 | 2.54E-49 | 2.17E-13 |
| CA12 | A member of zinc metalloenzymes that catalyze the reversible hydration of carbon dioxide. | 0.8 | 0.56 | -0.24 | -0.67 | 4.18E-50 | 3.82E-21 |
| S100A2 | Enhance P53 transcriptional activity | 0.85 | 0.62 | -0.23 | -0.65 | 8.68E-46 | 1.25E-20 |
| PKP1 | Mediates the interaction between desmosomal cadherin proteins with desmoplakin and keratin intermediate filaments. | 0.75 | 0.49 | -0.26 | -0.64 | 1.12E-43 | 9.49E-21 |
| ADH7 | A member of the alcohol dehydrogenase family. | 0.92 | 0.6 | -0.33 | -0.63 | 8.37E-43 | 2.63E-18 |
| RPL39L | Ribosomal protein 39 | 0.7 | 0.38 | -0.33 | -0.6 | 1.13E-37 | 8.77E-24 |
| ALDH3A1 | A metabolic enzyme that oxidizes toxic lipid peroxidation aldehydes to their corresponding carboxylic acids | 0.74 | 0.47 | -0.26 | -0.6 | 1.36E-37 | 9.54E-15 |
| TRIM29 | Negative regulator of the innate immune response to DNA viruses | 0.86 | 0.55 | -0.31 | -0.59 | 1.17E-36 | 2.09E-23 |
| EIF4EBP1 | Regulatory factor of the restriction process during translation initiation | 0.97 | 0.67 | -0.3 | -0.58 | 4.22E-35 | 1.55E-22 |
| CLDN1 | Responsibility for tight junctions in epithelial or endothelial cell sheets | 0.8 | 0.45 | -0.35 | -0.56 | 3.82E-32 | 1.11E-22 |
| KRT5 | An intermediate filament protein in the basal layer of stratified epithelial cells | 0.74 | 0.44 | -0.3 | -0.54 | 2.65E-29 | 4.09E-22 |
| IRF6 | An interferon regulatory factor | 0.92 | 0.6 | -0.33 | -0.53 | 3.01E-28 | 3.96E-19 |
| CSTA | Protein synthesis that contain multiple cystatin-like sequences | 0.72 | 0.33 | -0.39 | -0.52 | 4.06E-27 | 2.70E-19 |
| SLC7A11 | The functional subunit of system Xc- | 0.9 | 0.62 | -0.28 | -0.52 | 1.38E-26 | 2.00E-22 |
| LGALS7B | A member of β-galactoside-binding proteins modulating cell-cell and cell-matrix interactions. | 0.91 | 0.6 | -0.31 | -0.52 | 1.85E-27 | 1.58E-22 |
| CALML5 | Regulator of terminal epidermal differentiation genes and barrier function proteins | 0.81 | 0.57 | -0.23 | -0.52 | 2.53E-27 | 1.79E-21 |
| KRT6A | Encode the cytokeratin 6A protein. | 0.89 | 0.54 | -0.35 | -0.51 | 2.53E-26 | 2.09E-23 |
| DSG3 | A member desmoglein and cadherin cell adhesion molecule superfamily | 0.72 | 0.44 | -0.28 | -0.51 | 7.14E-26 | 4.31E-19 |
| CEP55 | Regulation of two daughter cells' physical disjunction | 0.91 | 0.55 | -0.36 | -0.5 | 5.15E-25 | 2.09E-23 |
| DSP | Maintain the mechanical integrity of epithelium | 0.84 | 0.48 | -0.35 | -0.5 | 8.24E-25 | 1.07E-23 |
| DSC3 | A principle component of desmosomes. | 0.97 | 0.75 | -0.22 | -0.5 | 1.18E-24 | 3.92E-22 |
| ITGB4 | Integrin associated transmembrane glycoprotein receptors. | 0.8 | 0.5 | -0.29 | -0.5 | 4.52E-25 | 6.06E-21 |
| TNS4 | Regulatory factor of cell adhesion | 0.76 | 0.47 | -0.28 | -0.49 | 3.09E-23 | 2.00E-22 |
| PHGDH | The first committed enzyme in the serine synthesis | 0.75 | 0.42 | -0.33 | -0.49 | 8.15E-24 | 7.24E-22 |
| HMGA1 | Regulate the interaction between the transcriptional regulatory proteins and downstream DNA | 0.83 | 0.55 | -0.28 | -0.48 | 1.82E-22 | 6.71E-24 |
| HJURP | Mediate CENP-A deposition at human centromeres during the early G1 phase | 0.62 | 0.39 | -0.23 | -0.47 | 3.86E-22 | 3.92E-22 |
| CYP4F3 | Regulator of drug metabolism and synthesis of cholesterol, steroids and other lipids. | 0.66 | 0.44 | -0.23 | -0.47 | 3.69E-22 | 7.62E-22 |
| SLC2A1 | Regulatory factor of energy metabolism | 0.89 | 0.49 | -0.4 | -0.46 | 2.34E-20 | 2.93E-24 |
| VSNL1 | Regulator factor of calcium-dependent and adenylate cyclase signal transduction | 0.74 | 0.45 | -0.29 | -0.45 | 6.35E-20 | 1.58E-21 |
| BIK | BH3-only pro-apoptotic protein | 0.96 | 0.68 | -0.28 | -0.43 | 6.81E-18 | 1.34E-20 |
| PRC1 | E3 ubiquitin ligase for histone H2A at lysine 119 | 0.48 | 0.2 | -0.28 | -0.41 | 1.69E-16 | 2.50E-22 |
| KRT15 | Responsibility for the structural integrity of epithelial cells | 0.66 | 0.39 | -0.28 | -0.4 | 2.25E-15 | 4.09E-22 |
| BMP7 | A secreted ligand of the TGF-β | 0.73 | 0.56 | -0.17 | -0.4 | 1.58E-15 | 1.38E-15 |
| SLC7A5 | A neutral amino acid transporter | 0.35 | 0.15 | -0.2 | -0.37 | 9.38E-14 | 3.16E-21 |
| EPN3 | Regulatory factor of receptor endocytosis | 0.75 | 0.42 | -0.34 | -0.37 | 3.76E-13 | 7.42E-23 |
| ARNTL2 | Regulatory factor of biologically relevant partner of circadian and hypoxia factors | 0.87 | 0.53 | -0.34 | -0.36 | 4.66E-13 | 6.71E-24 |
| KRT6B | Regulatory factor of monolayer and lamellar epithelium differentiation | 0.82 | 0.51 | -0.31 | -0.36 | 6.52E-13 | 1.17E-21 |
| NCAPH | Regulatory subunit of the condensin complex | 0.88 | 0.64 | -0.23 | -0.35 | 4.19E-12 | 7.51E-14 |
| GAPDH | Regulatory factor of glycolytic effects | 0.61 | 0.31 | -0.29 | -0.35 | 6.14E-12 | 2.09E-23 |
| GGH | Regulate intracellular folates and antifolates | 0.91 | 0.69 | -0.22 | -0.35 | 2.25E-12 | 4.73E-15 |
| CDK1 | Drive progression of cells through the entire cell cycle | 0.82 | 0.53 | -0.29 | -0.33 | 3.75E-11 | 2.62E-22 |
| PERP | Maintain the epithelial integrity and homeostasis | 0.75 | 0.47 | -0.28 | -0.32 | 3.18E-10 | 4.25E-20 |
