# Supplementary figures and images for: Identification and Validation of Three-Gene Signature in Lung Squamous Cell Carcinoma by Integrated Transcriptome and Methylation Analysis
Source: J Oncol. 2022 Sep 23;2022:9688040. doi: 10.1155/2022/9688040 (PMC9525794; doi:10.1155/2022/9688040)

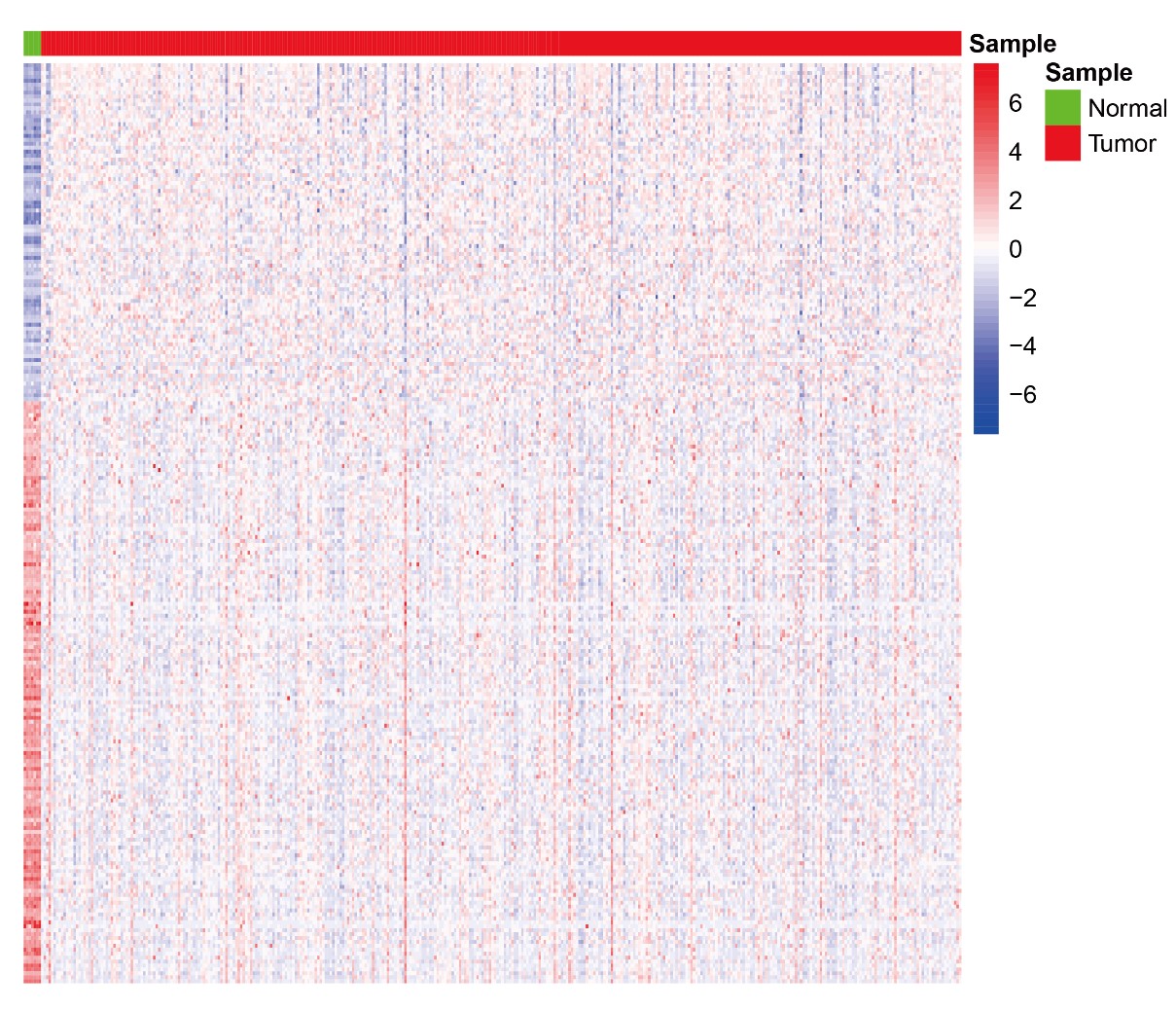

Supplement: Supplementary Materials — Figure S1. The heatmap for the DEGs between LUSC and paracancerous tissues. LUSC, Lung squamous cell carcinoma. Table SI. List of DNAm-driven genes in LUSC. [file 9688040.f1.zip › Figure S1.jpg]
